# Gene dosage-dependent rescue of HSP neurite defects in SPG4 patients’ neurons

**DOI:** 10.1093/hmg/ddt644

**Published:** 2013-12-30

**Authors:** Steven Havlicek, Zacharias Kohl, Himanshu K. Mishra, Iryna Prots, Esther Eberhardt, Naime Denguir, Holger Wend, Sonja Plötz, Leah Boyer, Maria C.N. Marchetto, Stefan Aigner, Heinrich Sticht, Teja W. Groemer, Ute Hehr, Angelika Lampert, Ursula Schlötzer-Schrehardt, Jürgen Winkler, Fred H. Gage, Beate Winner

**Affiliations:** 1 IZKF Junior Research Group and BMBF Research Group Neuroscience, IZKF, Friedrich-Alexander University Erlangen-Nuernberg (FAU), Glückstr. 6, Erlangen 91054, Germany; 2 Laboratory of Genetics, The Salk Institute for Biological Studies, 10010 North Torrey Pines Road, La Jolla, CA 92037, USA; 3 Department of Molecular Neurology and; 4 Department of Ophthalmology, Friedrich-Alexander University Erlangen-Nuernberg, Schwabachanlage 6, Erlangen 91054, Germany; 5 Institute of Physiology and Pathophysiology, Friedrich-Alexander University Erlangen-Nuernberg, Universitätsstraße 17, Erlangen 91054, Germany; 6 Institute of Biochemistry (Emil-Fischer-Zentrum), Friedrich-Alexander University Erlangen-Nuernberg, Fahrstraße 17, Erlangen 91054, Germany; 7 Department of Psychiatry, Friedrich-Alexander University Erlangen-Nuernberg, Schwabachanlage 6, Erlangen 91054, Germany; 8 Department of Human Genetics, Centre for Human Genetics, University of Regensburg, Franz-Josef-Strauss Allee 11, Regensburg 93053, Germany

## Abstract

The hereditary spastic paraplegias (HSPs) are a heterogeneous group of motorneuron diseases characterized by progressive spasticity and paresis of the lower limbs. Mutations in *Spastic Gait 4* (*SPG4*), encoding spastin, are the most frequent cause of HSP. To understand how mutations in SPG4 affect human neurons, we generated human induced pluripotent stem cells (hiPSCs) from fibroblasts of two patients carrying a c.1684C>T nonsense mutation and from two controls. These SPG4 and control hiPSCs were able to differentiate into neurons and glia at comparable efficiency. All known spastin isoforms were reduced in SPG4 neuronal cells. The complexity of SPG4 neurites was decreased, which was paralleled by an imbalance of axonal transport with less retrograde movement. Prominent neurite swellings with disrupted microtubules were present in SPG4 neurons at an ultrastructural level. While some of these swellings contain acetylated and detyrosinated tubulin, these tubulin modifications were unchanged in total cell lysates of SPG4 neurons. Upregulation of another microtubule-severing protein, p60 katanin, may partially compensate for microtubuli dynamics in SPG4 neurons. Overexpression of the M1 or M87 spastin isoforms restored neurite length, branching, numbers of primary neurites and reduced swellings in SPG4 neuronal cells. We conclude that neurite complexity and maintenance in HSP patient-derived neurons are critically sensitive to spastin gene dosage. Our data show that elevation of single spastin isoform levels is sufficient to restore neurite complexity and reduce neurite swellings in patient cells. Furthermore, our human model offers an ideal platform for pharmacological screenings with the goal to restore physiological spastin levels in SPG4 patients.

## INTRODUCTION

Hereditary spastic paraplegias (HSPs) are a genetically and clinically heterogeneous group of neurodegenerative disorders whose main clinical feature is progressive spasticity and paresis of the lower limbs (1). The main pathological characteristic is suspected to be an axonopathy of the longest corticospinal tracts and ascending dorsal columns, but there are only rare neuropathological studies on spinal cord tissue of HSP patients to clearly define structural changes relevant to the disease (2–4). HSPs have a prevalence between 5 and 10 per 100 000 and are autosomal dominant, autosomal recessive or X-linked. Forty percent of the dominantly inherited paraplegias with a pure HSP phenotype are due to mutations in *Spastic Gait 4* [*SPG4*, alternative Gene name *Spastic Gait* 4 (*SPAST*)] encoding spastin. More than 60 different mutations in *SPAST* have been reported so far (5–7). Although drugs, e.g. agonists for the gamma-aminobutyric acid type B receptors, symptomatically relieve spasticity, curative therapies and approaches to halt disease progression are completely lacking.

Spastin is a 616 amino acid protein of 67.2 kDa. To date, there are four known isoforms of the spastin protein, which are generated by an additional translation initiation site and differential exon splicing. These isoforms comprise a M1 full-length (616 amino acid) protein, a shorter isoform that lacks the first 86 amino acids of the full-length protein (M87) and splice variants of both of these, excluding exon 4 (M1ΔEx4, M87ΔEx4; altogether referred to as M1 and M87 isoforms hereafter) (8,9). Spastin is a member of the ATPases associated with various cellular activities (AAA) protein family. It is assumed, that adenosine triphosphate (ATP)-bound spastin forms hexameric rings with a central core, and it has been proposed that the positively charged central pore of spastin is able to pull the negatively charged C-terminal tail of tubulin and generate breaks in the microtubule (10,11). The spastin domains involved in microtubule severing are the microtubule-binding domain (residues 270–328) and the AAA domain (residues 342–599) (5).

Protein studies in rodents and humans showed spastin expression in different tissues, with abundance in the central nervous system (8,12,13). Very few postmortem analyses of HSP patients exist; one SPG4 patient showed corticospinal tract pathology with myelin pallor and loss of axons in the lateral and ventral corticospinal tracts (2–4,14).

Here, we generated a human neuronal SPG4 *in vitro* model derived from SPG4 patients' induced pluripotent stem cells and showed that the complexity of the neurite network was impaired in SPG4-derived neuronal cells. In addition, we provide evidence at an ultrastructural level that SPG4 neurites formed abundant and prominent swellings with severe disruptions of the microtubule system. Furthermore, we demonstrate that imbalanced movement of mitochondria might be implicated in HSP pathogenesis. Importantly, the neurite impairments were fully reverted by lentiviral overexpression of single spastin isoforms (M1 or M87), revealing for the first time that SPG4-related pathological phenotypes can be abrogated in patients' cells.

## RESULTS

### Generation of human induced pluripotent stem cell from SPG4 patients and controls

**Figure 1.**
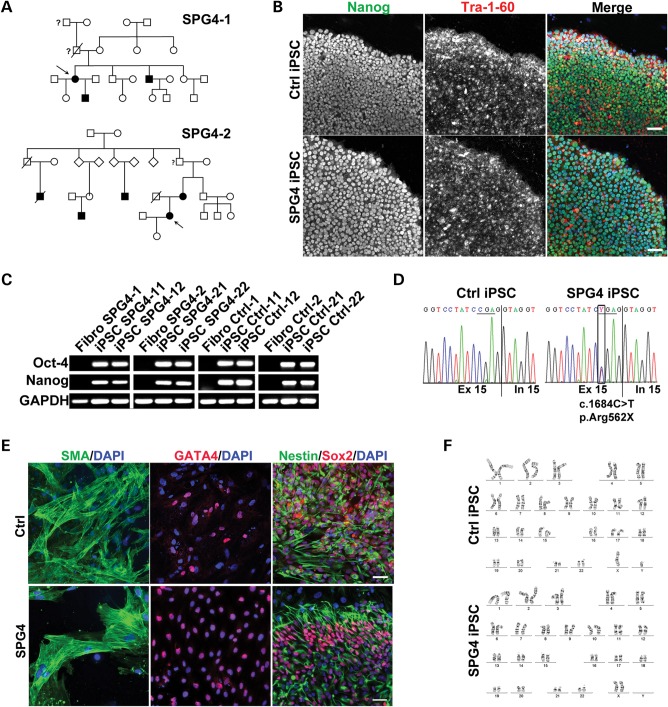
Pedigrees of SPG4 patients and characteristics of hiPSC. (**A**) Pedigrees of patients included in the study (arrows). (**B**) Patients' and control hiPSCs are morphologically similar to human embryonic stem cells, have well-defined borders and express the pluripotency-associated proteins Nanog and Tra-1-60. Representative images of Ctrl-11 and SPG4-21. (**C**) Patient and control hiPSCs but not fibroblasts express endogenous Nanog and Oct-4 transcripts as shown by RT–PCRs. (**D**) All patient hiPSCs were sequenced for the presence of the heterozygous SPG4 mutation c.1684C>T (p.R562X) as shown here for SPG4-21. (**E**) Undirected differentiation of all patient and control hiPSCs gave rise to progeny of all three germ layers. Representative images shown: SMA (mesoderm), Gata4 (endoderm) and Nestin/Sox2 double-positive cells (ectoderm). (**F**) All hiPSCs maintained a normal stable karyotype by G-banding analysis. Representative karyograms of Ctrl-11 and SPG4-21. Scale bars in (B) and (E) are 50 µm.

The control primary human fibroblast lines were established from healthy controls with no history of movement disorder or neurologic disease. They were age matched with two SPG4 patients who both carried the heterozygous SPG4 nonsense mutation c.1684C>T (p.R562X; pedigrees in Figure 1A and clinical information in Table 1). Fibroblasts from all subjects were infected with retroviral reprograming vectors (Klf4, c-Myc, Oct4, and Sox2), as previously described (15,16). After 2–3 weeks, compact human induced pluripotent stem cell (hiPSC) colonies emerged from a background of fibroblasts and were manually picked and cultured under feeder-free conditions. Two control (Ctrl)-hiPSC and SPG4-hiPSC lines from each subject were generated, which expressed endogenous pluripotency markers such as Nanog and Tra-1-60 (Fig. 1B, Supplementary Material, Fig. S1). Pluripotency was also confirmed by reverse transcription–polymerase chain reaction (RT–PCR) analysis for endogenous expression of Oct-4 and Nanog (Fig. 1C). The spastin mutation c.1684C>T was confirmed in the hiPSC clones from the SPG4 patients (Fig. 1D). Undirected *in vitro* differentiation contained derivatives from all three embryonic germ layers, which was confirmed by immunostaining for smooth muscle actin (SMA, mesoderm), GATA-binding protein 4 (Gata4, endoderm) and Nestin/Sox2 double-positive cells (ectoderm; Fig. 1E). All hiPSC lines used in this study maintained a normal karyotype (Fig. 1F), and PCR fingerprinting confirmed derivation from respective fibroblasts (data not shown). We will refer to hiPSCs as Ctrl-11/-12 (from Ctrl-1), Ctrl-21/-22 (from Ctrl-2), SPG4-11/-12 (from SPG4-1) and SPG4-21/-22 (from SPG4-2) hereafter.

**Table 1.** Clinical evaluation of patients and controls

	Sex	Mutation	Age onset/examination (years)	SPRS (max 52)	Landmark of disability	Barthel index	MMSE (30)	MRI (spinal/brain)
SPG4-1	F	R562X (Stop)	36/51	23	3	100%	30	Normal/normal
SPG4-2	F	R562X (Stop)	32/48	18	3	100%	30	n.d.
Ctrl-1	M	–	–/53	0	0	100%	30	n.d./normal
Ctrl-2	F	–	–/46	0	0	100%	30	n.d./normal

Patients’ (SPG4) and controls' (Ctrl) fibroblasts have been used in this study.

SPRS, spastic paraplegia rating scale (32); Barthel index, Barthel index of activity of daily living (max 100%); MMSE, mini-mental state examination; MRI, magnetic resonance imaging; n.d, not done.

### Generation of neurons from hiPSCs

**Figure 2.**
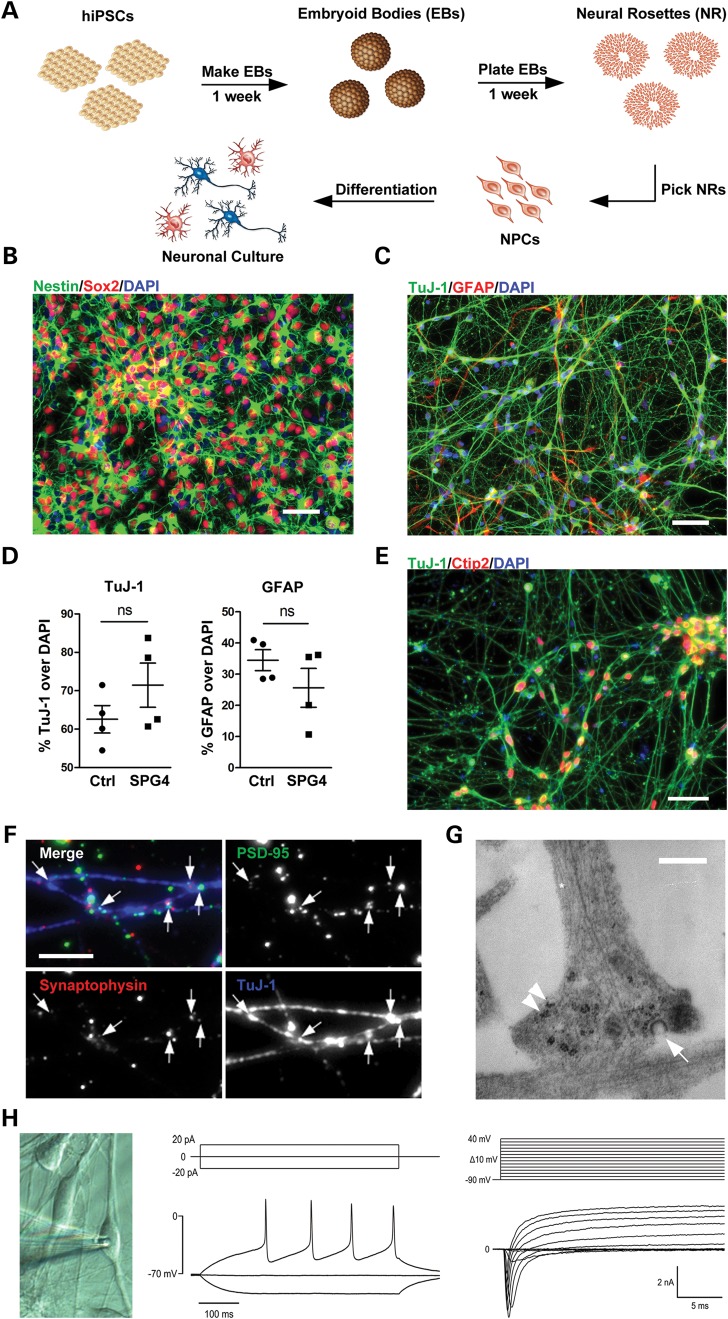
Generation and characterization of neuronal cultures. (**A**) The schematic view of the neural differentiation protocol. (**B**) Neural precursor cell (NPC) lines coexpress the early neural precursor markers Nestin and Sox2. (**C**) Neuronal cultures express neuronal (TuJ-1) and glial (GFAP) markers. (**D**) Statistical analysis for TuJ-1 and GFAP (% over DAPI) revealed no difference between patients and controls. Plotted are means of each line, *n* represents two independent experiments per line in triplicates. Data shown as mean ± standard error mean (SEM). (**E**) Staining for the cortical marker Ctip2 and TuJ-1 is shown. (**F**) Labeling for Synaptophysin and PSD-95 after 6 weeks of differentiation reveal punctuate staining in close proximity to crossing neurites indicating synapse formations. (**G**) Ultrastructural analysis further indicates synapse formation. Axon-like process containing microtubule structures (asterisk) forms a terminal button containing ribosomes (arrowheads) and vesicles (arrow). (**H**) Both, control and SPG4 differentiated neuronal cells display voltage-gated sodium and potassium channels (right column, voltage-clamp recordings) and fire action potentials (left column, current-clamp recordings, cells were held at −70 mV), reminiscent of neurons. Scale bars 50 µm in (B, C and E); 5 µm in (F) and 500 nm in (G).

Neural differentiation was initiated by generating free-floating embryoid bodies (EBs), which were plated on a laminin-coated surface. Neural rosettes were manually collected, dissociated and replated as proliferative neural precursor cells (NPCs), which coexpressed the early neural precursor markers Nestin and Sox2 (Fig. 2A and B). We established two NPC lines from each patient (SPG4-111, -121, -211, -212) and control (Ctrl-111, -121, -211, -212). We did not detect any obvious differences in Nestin/Sox2 double-positive cell numbers and proliferation between SPG4 and control NPCs (data not shown). After terminal differentiation, the cells were analyzed for TuJ-1 (b-III-tubulin, neuronal marker) or GFAP (astroglial marker) expression (Fig. 2C). The percentages of TuJ-1- and GFAP-expressing cells were not different between patients and controls (percent over DAPI; *P* = 0.24 and 0.26, respectively, Fig. 2D, and Supplementary Material, Fig. 2A), indicating that patient and control hiPSCs were able to form NPC lines giving rise to neurons and glia with variation between lines but no significant difference between SPG4 and controls.

Our hiPSC-derived neuronal cells expressed axonal and dendritic markers like the microtubule-associated proteins Tau, and Map2a/b, were mostly glutamatergic (Supplementary Material, Fig. 2B and D), and mostly stained positive for the cortical marker COUP TF-interacting protein 2 (Ctip2; Fig. 2E, 72.17 ± 14.23% over TuJ-1), which is expressed by cortical layer 5/6 neurons *in vivo*, including corticospinal motorneurons (17). Synaptophysin- and postsynaptic density 95 (PSD-95)-positive puncta on neurites indicated formation of synapse-like structures (Fig. 2F). Synapse formation was further supported by transmission electron microscopy showing axon-like processes that formed vesicle-filled terminal buttons (Fig. 2G). In addition, 95%/100% (disease/control) of the tested cells displayed voltage-gated ion channels such as sodium and potassium channels, and 87%/88% generated action potentials or spiklets, suggesting the presence of functional neuron-like cells (Fig. 2H).

### Differential expression of spastin in SPG4 patients

**Figure 3.**
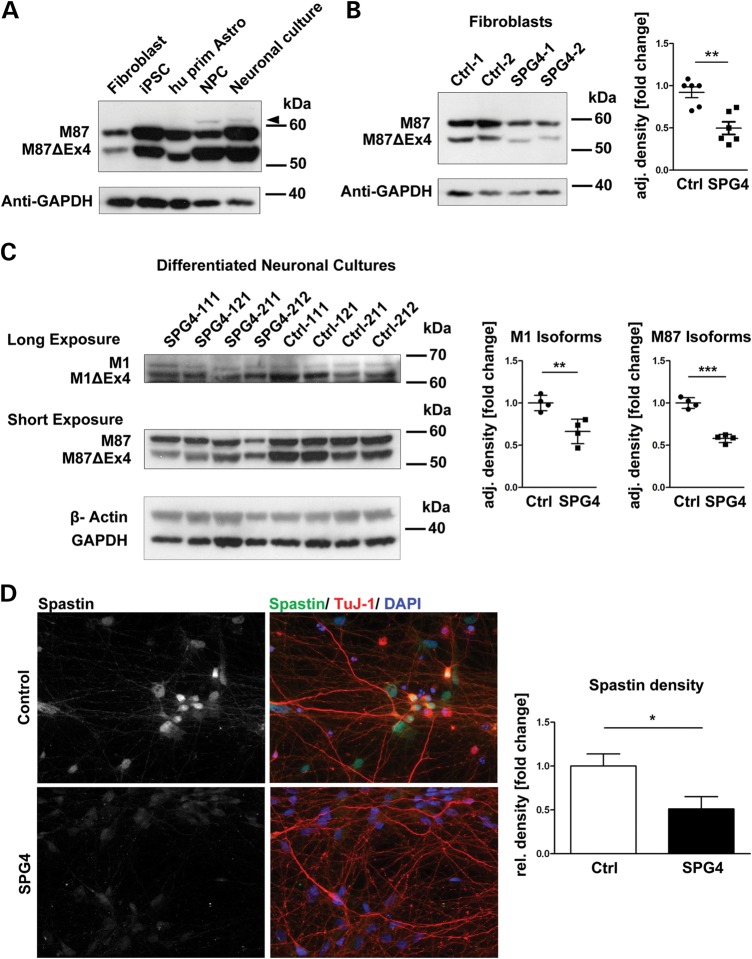
Differential expression of spastin isoforms in SPG4 patients. (**A**) Comparison of spastin expression in different human cell types by western blotting: Hu prim Astro = human primary cerebellar astrocytes, NPC = hiPSC-derived NPCs. The M87 and M87ΔEx4 isoforms are the predominant bands. It is noteworthy that only NPCs and neuronal cultures, but not astrocytes or non-neuronal cells, show expression of isoforms bigger than 60 kDa (faint bands). (**B**) Significant reduction in spastin protein levels is already evident in fibroblast cultures from patients. Right image shows the fold changes in the adjusted density of spastin (normalized against GAPDH). *n* = 3 replicates, data shown as means ± SEM. (**C**) Neuronal cultures show a reduction in expression of the M87 isoforms (short exposure, middle panel, 58% of M87 isoforms (*P* ≤ 0.0001) and the M1 isoforms [longer exposure, top panel, 66% M1 isoforms (*P* = 0.004)], in patient samples. Protein amounts loaded on gels are 20 µg per lane. (**D**) Imaging of endogenous spastin levels in neuronal cultures revealed an average reduction in spastin to 50.3% in SPG4 samples. *n* = 2 per line. Data shown as means ± SEM.

Little is known about the expression of spastin and its isoforms in different human cells. We analyzed the expression in our control primary fibroblasts, hiPSCs, NPCs, neuronal cultures and human primary astrocytes (Fig. 3A). We observed that the two major isoforms present in every cell type were M87 and M87ΔEx4. Notably, expression levels of spastin were increased in NPCs and neuronal cultures and, to a lesser extent, in hiPSCs. The presence of M1 isoforms was restricted exclusively to NPCs and neuronal cultures (Fig. 3A and C).

We further sought to determine whether the levels of spastin protein in SPG4 patients were altered, and observed that the spastin M87 isoforms were significantly reduced by 48% in SPG4 fibroblasts (*P* = 0.002; Fig. 3B). In hiPSC-derived neuronal cultures, we observed M1 isoforms (M1 and M1ΔEx4) and M87 isoforms (M87 and M87ΔEx4) in control and SPG4 lines. The levels of M1 isoforms and M87 isoforms were significantly decreased in SPG4 patients' neuronal cultures (34%, *P* = 0.008, and 40%, *P* < 0.0001, reduction in M1 and M87 isoforms, respectively; Fig. 3C). To further validate this finding, we performed density measurements on spastin stainings of our neuronal cultures and confirmed a significant decrease in spastin protein level in SPG4 patients (49.7% decrease on average, *P* = 0.024, Fig. 3D).

The premature termination codon of the c.1684C>T nonsense mutation is within the AAA ATPase domain, and therefore transcripts of this allele are expected to induce nonsense-mediated mRNA decay. Consistent with this, we did not observe a truncated version in any SPG4 patient-derived cell type (fibroblasts, hiPSCs, NPCs and neuronal cultures) by immunoblot analysis (Fig 3B and C; data not shown). To validate that the antibodies used would be able to recognize truncated spastin, we introduced the p.R562X stop mutation into M1 and M87 spastin expression vectors and overexpressed them in HEK 293 T cells (Supplementary Material, Fig. 3).

### Morphological alterations in spastin neurons

**Figure 4.**
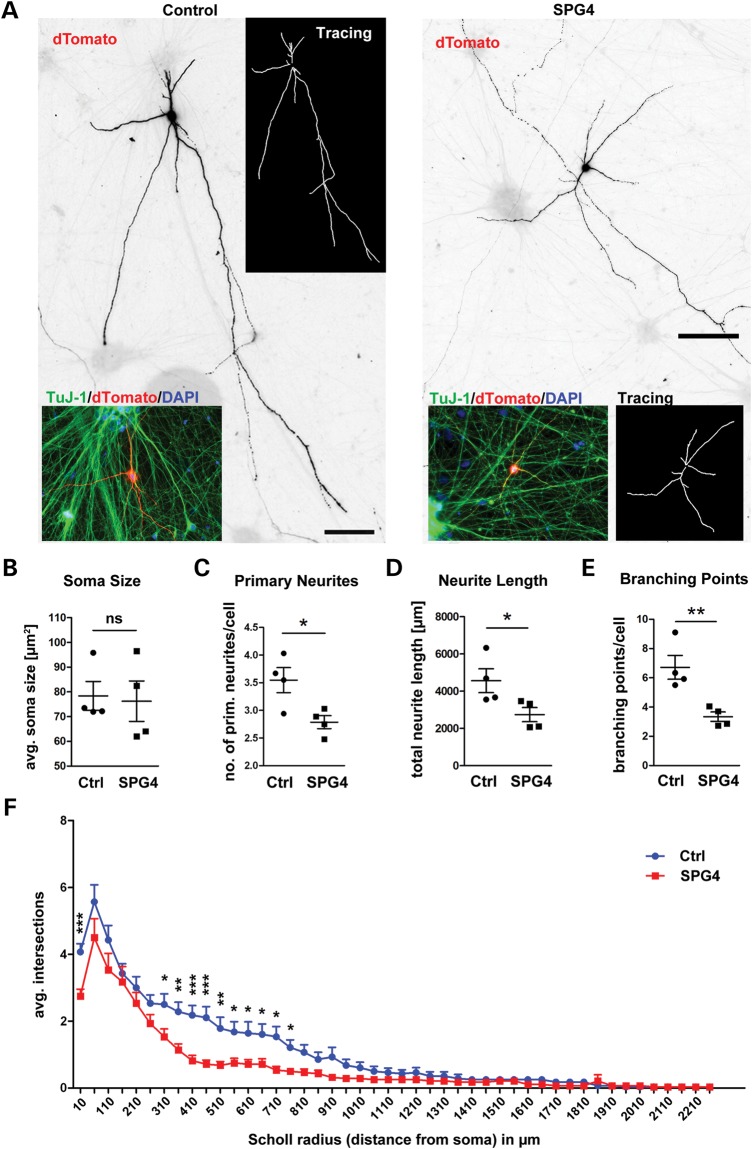
Significant decrease in neurite length and branching points in SPG4 neurons. (**A**) Neuronal cultures were transfected with pEF1-dTomato to visualize individual cells. Representative images of transfected neuronal control (left) and patient (right) neurons. Neurite tracings are shown in white inserts. (**B**) No difference in the average soma sizes between control and SPG4. *n* = two experiments in triplicates, data shown as means ± SEM. (**C**) The average number of primary neurites per cell is significantly decreased. (**D**) The average neurite length is significantly decreased in SPG4 neurons. (**E**) The average number of branching points is significantly decreased in SPG4 neuronal cultures. (C–E) Evaluation of transfected cells shown in (A). *n* = minimum of 21 cells per line with a total of 96 patient cells and 116 control cells from 4 to 6 wells. (**F**) Scholl analysis revealed a significant reduction in branching, especially in the proximal regions close to the soma (radii 310–860 µm) in SPG4 neurons and confirmed the reduction in primary neurites (10 µm radius). *N* = 20 cells per group. Data shown as means ± SEM.

Next, we investigated whether altered spastin levels might have an impact on human neurite morphology. To visualize processes of single neuronal cells within a dense network, we transfected the cultures with the pEF-1 dTomato construct described earlier (18) (Fig. 4A). While soma size was not changed between controls and SPG4 patients' neuronal cells (*P* = 0.153 Fig. 4B), the morphological analysis revealed that SPG4 neurons had significantly less primary neurites (*P* = 0.025, Fig. 4C), and total neurite length was significantly decreased by an average of 40% (*P* = 0.025, Fig. 4D). In addition, the number of branching points was significantly decreased by 50% (*P* = 0.004, Fig. 4E), indicating reduced neurite complexity of SPG4 neuronal cells. Scholl analysis of neurite complexity independently confirmed these findings and indicated that branching in regions proximal to the soma is most severely affected (Fig. 4F).

**Figure 5.**
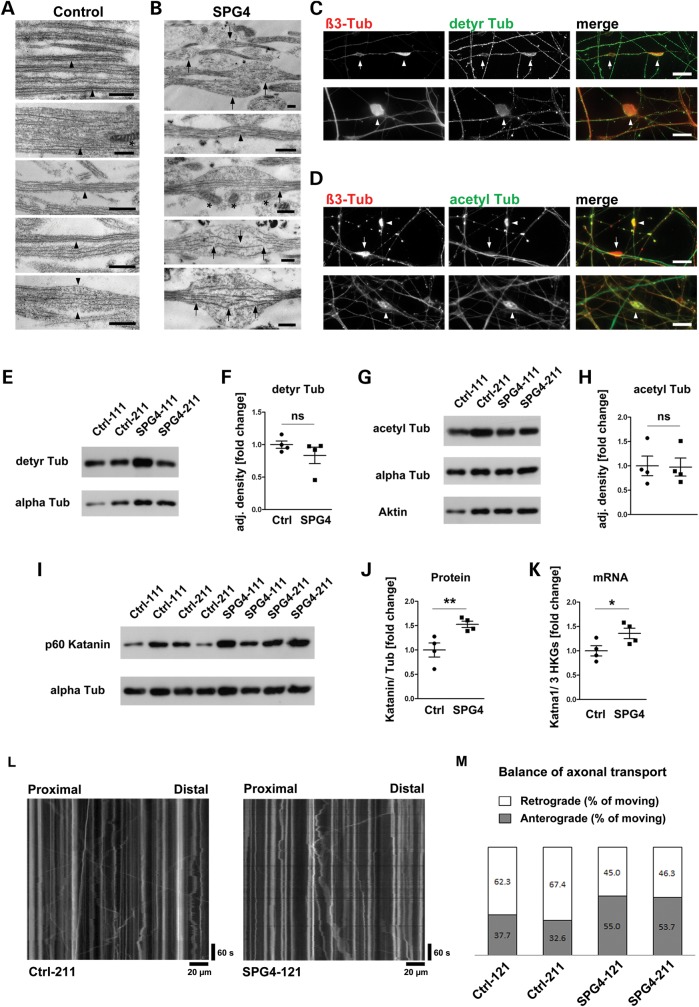
Ultrastructural and biochemical analyses of neurite swellings in SPG4 lines. (**A**) Multiple neurites showing regularly organized microtubule structures (arrowheads) and occasional mitochondria (asterisk in second panel) in control cells. Rarely, control neurites show mild focal bulging of the plasma membrane (arrowheads in bottom panel). (**B**) Most of SPG4 neuronal cells display prominent neurite swellings with disrupted microtubules (arrows) and occasional accumulation of mitochondria (asterisks in middle panel). Rarely, neurites only containing regularly organized microtubule structures are present in SPG4 lines (arrowhead in second panel). (**C**) The majority of swellings (arrowheads) did not stain for detyr Tub. Some swellings were devoid of tubulin staining (arrow). (**D**) The majority of swellings (arrowheads), but not all (arrow), stained intensely for acetylated tubulin (acetyl Tub). (**E**) Representative western blot of detyr Tub levels in two control and two SPG4 neuronal cultures. (**F**) Quantification of detyr Tub levels normalized against total alpha Tub levels revealed no difference in all four SPG4 lines compared with all four controls. (**G**) Representative western blot of acetyl Tub levels in two control and two SPG4 neuronal cultures. (**H**) Quantification of acetyl Tub levels normalized against total alpha Tub levels revealed no difference in all four SPG4 lines compared with all four controls. (**I**) Representative western blot of p60 katanin levels in control and SPG4 neuronal cultures. (**J**) Quantification of p60 katanin levels normalized against total alpha Tub levels revealed a significant increase in all SPG4 lines compared with controls (*P* = 0.008). (**K**) qRT–PCRs confirmed upregulation of Katna1 (gene encoding p60 katanin) mRNA levels in all SPG4 lines. mRNA levels were normalized against three housekeeping genes (HKGs = HPRT, GAPDH and β2M). Scale bars 500 nm (A and B) and 10 µm (C and D). (**L**) Representative kymographs of axonal mitochondria transport in Ctrl and SPG4. (M) Balance of anterograde and retrograde transport events in Ctrl and SPG4 lines (*P* = 0.007, *χ*^2^ test). Western blot data quantified from at least triplicates of two independent differentiations each. Data shown as means ± SEM.

To comprehensively analyze neurite processes in SPG4 lines, we performed ultrastructural analysis using transmission electron microscopy. In control cultures, we mostly observed bundles of straight and elongated neurites containing parallel arrays of microtubules and occasionally interspersed mitochondria, but only rare signs of moderate neurite swellings (Fig. 5A). In contrast, the neurites of SPG4 patients displayed abundant neurite swellings with loosely arranged, interrupted microtubules and occasionally accumulated mitochondria, but only rarely SPG4 neurons had neurites with a completely intact microtubule system (Fig. 5B). Taken together, these ultrastructural analyses imply that the maintenance of neuritic microtubule structures is severely impaired in SPG4 neuronal cultures. However, these neurite swellings did not affect the survival rate of SPG4 neurons as determined by viability (Supplementary Material, Fig. 4A and B) and apoptosis assays (Supplementary Material, Fig. 4C), recapitulating HSP pathogenesis, which is commonly not associated with cell death (reviewed in 1,19).

Previously, it had been suggested that neurite swellings are associated with increased stabilization of microtubules (2,20). Markers for acetylated and detyrosinated tubulin (detyr Tub), commonly referred as modifications of stabilized microtubules, were present in some, but not all, swellings of SPG4 neurons. While detyr Tub was rarely enriched in swellings (Fig. 5C), acetylated tubulin mostly showed a prominent staining (Fig. 5D). Some swellings were devoid of acetylated or detyr Tub (arrows in Fig. 5C and D). However, high levels of modified tubulins also correlated with enriched total tubulin levels in swellings, suggesting a general accumulation of tubulins in most swellings rather than a specific increase in the ratio of tubulin modifications. This led us to investigate the relative amounts of detyrosinated and acetylated tubulin normalized against total tubulin levels by western blotting in whole-cell lysates (Fig. 5E and G). No differences in total tubulin detyrosination or acetylation levels were present (*P* = 0.137 and 0.466, respectively; Fig. 5F and H).

Considering that spastin has a critical role in microtubule dynamics, it was surprising that there were no changes in stabilized tubulin levels. To further analyze potential underlying mechanisms, we investigated p60 katanin, another microtubule-severing enzyme. Strikingly, p60 katanin was significantly increased in all SPG4 lines on mRNA and protein expression levels (Fig. 5I–K), indicating that SPG4 neuronal cells at least partially compensate for loss of spastin by upregulating p60 katanin, which might help to keep the microtubules dynamic.

### Axonal transport imbalance in SPG4 neurons

To investigate whether the altered morphology of the SPG4 neurites has an impact on axonal transport, we performed time-lapse microscopy imaging. To unambiguously distinguish between antero- and retrograde transport events in axons, we differentiated our neuronal cultures in microfluidic chambers, where neurons project their axons from the cell soma side through narrow, 450 µm-long grooves to the opposite (axonal) chamber side. Importantly, glial cells were unable to project through the grooves (data not shown), allowing us to selectively investigate axonal transport in neurons. Lentivirus-mediated overexpression of Mito-DsRed visualized the mitochondria. After differentiation, when the axons had projected through the grooves, we investigated Mito-DsRed-positive mitochondria in one line per patient and control by time-lapse microscopy imaging, >20 neurites per line over 10 min, cumulating in 2116 Ctrl mitochondria and 1741 SPG4 mitochondria analyzed (Fig. 5L). In SPG4 neurons, the motility of mitochondria (amount of actively transported mitochondria) was unchanged (SPG4: 23.5 ± 0.58%, Ctrl: 19.1 ± 0.52%, *P* = 0.487).

Strikingly, we measured a significant imbalance in transport direction in SPG4 cells, which had reduced percentages of retrograde transport events and more anterograde transport events. On average, 64.9 ± 3.6% of mitochondria were transported retrogradely in control neurons compared with 45.7 ± 0.9% in SPG4 neurons (*P* = 0.007, Fig. 5M).

The mean velocities of anterograde (Ctrl: 0.274 ± 0.019 µm/s, SPG4: 0.296 ± 0.017 µm/s, *P* = 0.398) and retrograde (Ctrl: 0.374 ± 0.019 µm/s, SPG4: 0.373 ± 0.021 µm/s, *P* = 0.97) transport were unaltered. Similarly, the frequency distribution of speed ranges was unchanged (data not shown), suggesting no general impairment of initiated transport processes, but an imbalance in transport directions.

### Rescue of neurite defects by lentiviral overexpression of M1 or M87 spastin

**Figure 6.**
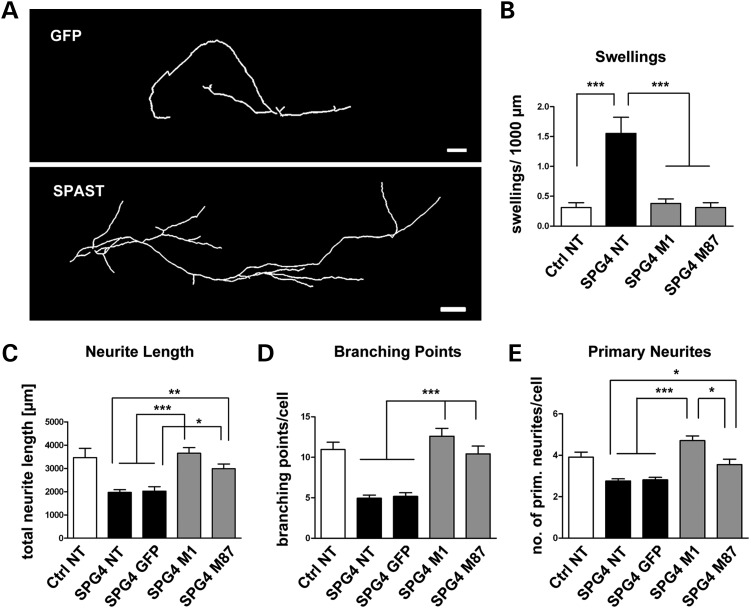
Reduction in neurite swellings and restoration neurite complexity by lentiviral overexpression of M1 or M87 SPAST. (**A**) Tracings of cells overexpressing GFP or the full-length M1 isoform of spastin after lentiviral transduction with an empty vector or by a pCAG-SPAST(M1)-IRES-GFP/or pCAG-SPAST(M87)-IRES-GFP vector, respectively. (**B**) Overexpression of M1 or M87 spastin reduces the number of swellings to control levels. (**C**) Spastin, but not GFP, overexpression restores the average neurite length to control levels. (**D**) Spastin, but not GFP, overexpression restores the average numbers of branching points per cell to control levels. (**E**) Spastin, but not GFP, overexpression significantly increases the average number of primary neurites per cell. There are significantly more primary neurites following M1 spastin overexpression compared with M87 spastin. (B–E) Evaluation of transduced cells shown in (A). NT, non-transduced; *n*, minimum of 20 cells per condition of 4–6 wells. Data shown as means ± SEM. Scale bars are 100 µm.

To directly link the observed disease phenotypes to the reduction in spastin levels, we asked whether the neurite defects might be reduced by elevation of spastin levels in the SPG4 neurons. We overexpressed SPAST(M1)-internal ribosome entry site (IRES)-green fluorescent protein (GFP) or SPAST(M87)-IRES-GFP, two lentiviral constructs, which, when overexpressed, produced a single M1 or M87 isoform band in western blots, respectively (Supplementary Material, Fig. 3). We used Ctrl neurons, non-overexpressing SPG4 neurons and GFP-overexpressing SPG4 neurons as controls. Importantly, overexpression of either of the spastin isoforms led to a reduction in neurite swellings and the restoration of the number of primary neurites, neurite length and branching points in SPG4 neuronal cultures [multiplicity of infection (MOI): 0.25–0.5, *P* ≤ 0.05 for all analyses, Figure 6A–D]. This indicates that the applied level of spastin overexpression led to an abrogation of the described neurite phenotype defects. We did not observe any effect of overexpression of spastin on control neurons under the same conditions (data not shown). Interestingly, an MOI of 1 or higher of SPAST-virus, but not GFP virus alone, was highly toxic for SPG4 and control neurons (data not shown), suggesting that the effect of spastin on human neurons is gene dosage-dependent.

## DISCUSSION

One of the most challenging aspects of spastin-related pathogenesis is that, despite its ubiquitous presence in many different organs, mutations of this protein result in the specific degeneration of processes of long-range projection neurons. This phenotypic specificity led us to develop a human neuronal *in vitro* model for HSPs using hiPSCs derived from SPG4 patients. We show here that neurons from SPG4 patients expressed reduced levels of spastin. Although they were capable of functionally maturing into neurons *in vitro*, total neurite length and arborization were decreased in SPG4 neuronal cells, indicating an altered neurite complexity, which was fully restored by moderate elevation of spastin levels. Changes in neurite morphology in neuronal cells from SPG4 patients are further supported by an ultrastructural analysis showing numerous neurite swellings and severely disrupted microtubule structures, which partly accumulated tubulin. However, the amount of acetylated and detyr Tubs within the cells was unaltered, and this might be explained by an upregulation of p60 katanin, which is known to play an important role in microtubule dynamics in neurons (21,22). A prominent imbalance of mitochondria distribution toward less retrograde transport was present.

Previously, only a few human analyses were performed on spastin expression patterns, some reporting neuronal expression of spastin not only in the motor cortex, but widespread in the brain and spinal cord (12,13). Our studies expand the expression of spastin to human fibroblasts, hiPSCs and astrocytes and indicate that the M87 isoforms of spastin are expressed in diverse human cell types. We detected the full-length M1 and M1ΔEx4 isoforms specifically in human neuronal cells. Similar protein expression patterns have been described for rodent tissues (8,23). Taking into account that the sole cell type affected in HSP is neurons, one might speculate that specifically the M1 isoforms have a crucial role in neuron integrity and/or survival, and in particular a reduction in these M1 isoforms is relevant for disease manifestation. However, our data show that the overexpression of either M1 or M87 isoform was sufficient to restore the neurite complexity and reduce swellings in SPG4 neuronal cells.

The p.R562X mutation of spastin was first described in a screen for the spectrum of SPG4 mutations and predicted to be a nonsense mutation (24). We never observed abnormally truncated versions of spastin in any SPG4 patient-derived cell line, most likely due to nonsense-mediated mRNA decay of transcripts carrying the mutation (25,26). The absence of truncated spastin in human SPG4 neurons convincingly demonstrates that haploinsufficiency is the leading cause of c.1684C>T SPG4-related HSPs in humans. However, other mutations might have a dominant negative effect (27).

We detected a significant decrease of 34% in M1 and 40% in M87 isoforms in SPG4 neuronal cultures by western blotting and up to 50% by immunofluorescence density measurements. Interestingly, the decrease in fibroblasts (∼48% decrease in M87 isoforms) was even more pronounced than in neuronal cells, indicating a potential role for patient-derived fibroblasts as a powerful pharmacological screening tool in future. A reduction in spastin expression levels in olfactory-derived CD105+ progenitors from SPG4 patients and hiPSC-derived neuronal cultures from one single-SPG4 case has been observed previously and is in line with our findings (28,29).

It is important to note that the quantitative decrease in spastin protein is less severe in neuronal cells, but causal for the specific disease phenotype related to long projecting neurons. This finding further emphasizes that human neurons are highly vulnerable to reduced levels of spastin. Interestingly, the level of spastin expression also correlated with neurite lengths in the SPG4 lines (e.g. the ‘high expresser’ SPG4–111 line—∼75% of wild-type spastin expression—also had the longest neurite length among our SPG4 lines, while the line with the least detectable spastin levels, SPG4-212, also had the shortest neurites). This finding indicates that the maintenance of human neurites is critically associated with the fine-tuned expression levels of spastin.

It is well accepted that one of the main functions of spastin is to sever microtubules and that it plays an important role in microtubule dynamics (10,11,30). We therefore hypothesized that alterations in the microtubule network might be a consequence of decreased spastin levels in the SPG4 neurons and this might lead to structural defects. We comprehensively analyzed cell morphology in our SPG4-derived neuronal cultures by tracing individual neurons within a dense network of cells after transfection with a fluorescence reporter construct and determined that they had less primary neurites and that those neurites were significantly decreased in length and branching, indicative of decreased neurite complexity. This phenotype was independently confirmed by applying Scholl analysis of neurite complexity on a different batch of neuronal cultures. In drosophila, regeneration of an axon from the axon stump after distal axotomy has been shown to be sensitive to spastin gene dosage, as loss of one copy of the drosophila homolog D-spastin gene impaired this process (30). Interestingly, axonal outgrowth studies in zebrafish, and spastin knockdown in rodent hippocampal cultures have also suggested that spastin is required for normal neuronal outgrowth during development (21,31,32). In conjunction with these models, our human SPG4 model highlights for the first time that spastin is one of the key regulators of neurite *de novo* initiation, outgrowth and neurite regeneration in humans.

Our ultrastructural analysis revealed axonal swellings in heterozygous SPG4 patient-derived neurons. Importantly, the reduction in human spastin by only 30–50% led to a pronounced phenotype with abundant neurite swellings in most neurons analyzed. Only rare swellings were observed in cortical neuronal cultures from homozygous knockout mice, completely lacking spastin (SpastΔE7 and SpastΔE5-7) (2,20). In contrast to our findings in humans, the number of swellings was very low in these mice (4 swellings per 100 cells analyzed) (20). Furthermore, the knockout mice show very subtle behavioral phenotypes with largely unaffected locomotion (2,20). Both structurally and behaviorally, humans are much more severely affected by altered spastin levels, indicating that human neuronal cells are more sensitive to spastin dosage changes than murine neurons are.

We show here that some swellings are enriched for stable microtubules and these swellings also stain positive for increased total tubulin, but these subtle differences do not reflect on differences of total acetylated and detyr Tub in western blots of whole-cell lysates, when normalized to total tubulin. Previous reports had suggested that increased stabilization of microtubules was associated with neurite swellings (2,20,29); however, these studies did not look at total tubulin levels in their samples, and without normalization to a reference point, conclusions drawn from these studies are to be considered with caution. Interestingly, our results indicate that microtubule dynamics may be maintained in SPG4 neurons by endogenous upregulation of the microtubule-severing enzyme p60 katanin. p60 katanin has been shown to have a critical role in regulating microtubule dynamics during neuronal outgrowth (22,33). Since this increase in the katanin level is not able to rescue the neuritic phenotypes observed in patients, there may be specific functions of spastin and/or specific locations of spastin that cannot be substituted for by other severing enzymes. Future work will have to address the minute differences between these two severing enzymes in human neurons.

Alterations in axonal transport are discussed to be a common pathological mechanism not only in several HSPs but also in other neurodegenerative diseases (34). We extensively analyzed mitochondrial transport in neuronal cultures of our patient and control lines. We revealed a relative decrease in reterograde axonal transport, implicating an imbalance in cargo distribution caused by reduced levels of spastin.

Interestingly, motility and mean velocity as well as frequency distribution of the cargo speeds were unaltered in SPG4 neurons. While this report was in preparation, another group recently reported axonal transport alterations in one neuronal culture from a single-SPG4 patient when compared with one control culture (29). Although the authors claim an overall reduced motility of mitochondria, contrasting to our findings, they specifically find retrograde transport events to be affected, which is in line with our observations. The differences in mitochondria motility might be because of the numbers of replicates and/or assay setup.

The observed neurite swellings and the structural alterations of cytoskeletal components therein described in our human SPG4 model are indicative of degenerative processes in patients’ neurons. These phenotypes might represent early stages of the dying back axonopathy seen in patients, proving the usefulness of our hiPSC-derived SPG4 model for investigating HSP disease mechanisms. Strikingly, we were able to restore neurite length and branching, as well as the number of primary neurites by overexpression of either M1 or M87 spastin, indicating that restoring spastin to more physiological levels is crucial for neurite development and maintenance. The abrogation of neurite defects by elevating M1 or M87 spastin levels in the neuronal cultures further indicates that the mutation is unlikely to have a dominant negative effect.

In conclusion, our data are consistent with a gene dosage-dependent neurodegenerative effect of spastin in human SPG4 neurons and suggest that bringing wild-type spastin expression back to normal levels might halt neuronal degeneration in patients. Our human *in vitro* model provides the foundation for compound screenings with the goal to restore physiological spastin levels in patient samples and thereby paves the road to discover new treatment strategies for SPG4-related HSPs.

## MATERIALS AND METHODS

### Patients and fibroblast derivation

The patients included (*n* = 2) were Caucasians with prototypical characteristics of spastic paraplegia and genetically confirmed heterozygous mutations at position c.1684C>T/p.R562X in the *SPAST* gene (*SPG4*) (35). The controls (*n* = 2) were healthy Caucasian individuals with no history of movement disorder or neurologic disease. The detailed clinical and genetic characteristics are summarized in Table 1. The human fibroblasts were obtained from dermal punch biopsies of the upper arm as previously described, following Institutional Review Board approval (no. 4120: *Generierung von humanen neuronalen Modellen bei neurodegenerativen Erkrankungen*) and informed consent at the movement disorder clinic at the Department of Molecular Neurology, Universitätsklinikum Erlangen (Erlangen, Germany). Fibroblasts were cultured in IMDM/Glutamax containing 15% fetal bovine serum (FBS), (Invitrogen) and 1× penicillin/streptomycin (all Invitrogen).

### hiPSC derivation

Low passage fibroblasts seeded in a six-well plate (Corning) in IMDM/Glutamax and 15% FBS (both Invitrogen) were infected twice daily for 2 days with equal amounts of supernatants of Sox2-, Klf4- and c-Myc-containing retrovirus and three times the amount of Oct3/4 by spinfection (800 g for 60 min; 4 µg/µl Polybrene) (15,16,36). One day after infection, fibroblasts were detached using TrypLE Express cultured with hESC medium: DMEM/F12/Glutamax, 20% Knockout Serum Replacement, 1× NEAA (all Invitrogen), 55 µM β-Mercaptoethanol (Sigma-Aldrich), 10 ng/ml fibroblast growth factor 2 (FGF2) (Preprotech) and 10 µM SB431542 (Sigma) on a feeder layer of irradiated mouse embryonic fibroblasts (Millipore). HESC medium was changed every other day. hiPSC colonies started to appear ∼Day 12 after infection and were manually isolated under a stereo microscope (Olympus) and transferred to feeder-free conditions on 24-well plates (Corning) coated with 0.5 mg Matrigel (BD Biosciences) in mTeSR1 medium (Stemcell Technologies). hiPSCs were expanded clonally and passaged every 7–8 days with Collagenase IV (200 U/ml), and a manual disintegration using 1 ml glass pipettes.

### Neuronal and spontaneous differentiation

We generated EBs by transferring hiPSCs to ultralow attachment plates (Corning) in mTeSR1 (Stemcell Technologies). For directed neuronal differentiation, the EB colonies were maintained in suspension in N2/B27 medium: DMEM/F12/Glutamax, with N2 and B27 (w/o VitA) supplements and Pen/Strep (all Invitrogen) from Days 2–7 and then plated onto polyornithine (PORN)/laminin (Invitrogen) coated plates. Visible rosettes formed within 1 week and were manually dissected under a stereo microscope (Olympus) and cultured in N2/B27 medium and 20 ng/ml FGF2 (Preprotech) to form proliferative NPC lines. NPCs were maintained at high density, grown on PORN/laminin-coated plates in N2/B27 medium and split ∼1:3 every 5–10 days with TrypLE Express (Invitrogen). Terminal differentiation of NPCs was initiated in neural differentiation medium (N2/B27 medium, 20 ng/ml brain derived neurotrophic factor, 20 ng/ml glial cell line-derived neurotrophic factor (both Peprotech), 1 mm dibutyryl-cyclic adenosine monophosphate, 200 nM ascorbic acid (both Sigma) at a density of ∼40 000 cells/cm^2^ on PORN/laminin-coated plates or glass coverslips (Thermo Scientific). Neurons were cultured under these conditions for 4–8 weeks with a half medium change every week.

For spontaneous undirected differentiation, hiPSCs were differentiated to form progeny of all three germ layers by forming EBs in FBS-containing medium: IMDM/Glutamax, 10% FBS (both Invitrogen). After 2 weeks, EBs were manually disrupted by pipetting and cultured for 1–3 weeks on 0.1% gelatin (Sigma-Aldrich)-coated plates (for RNA isolation) or PORN/laminin-coated glass coverslips (for immunofluorescence staining). Human primary cerebellar astrocytes were obtained from ScienceCell (#1810) and cultured according to producer’s instructions.

### Immunofluorescence staining

Cells were fixed in 4% paraformaldehyde in phosphate buffered solution (PBS) (Invitrogen) at room temperature (RT) for 10–20 min and permeabilized and blocked at RT for a minimum of 30 min in PBS++ (0.3% Triton X 100 (Sigma) in PBS (Invitrogen) and 3% donkey serum (Sigma)). Primary antibodies were incubated overnight at 4°C in PBS++. Secondary antibodies were incubated for 2–3 h at RT in PBS++. Coverslips were mounted on glass microscope slides (Thermo Scientific) in Aqua Polymount (Polysciences). Images were acquired using a fluorescence microscope (Observer.Z1, Zeiss). Primary and secondary antibodies are listed in Supplementary Material, Table S1. To visualize nuclei, 0.5 mg/ml DAPI (49,6-diamidino-2-phenylindole) was used.

### Transmission electron microscopy

Transmission electron microscopy was performed as previously described (37). In brief, neuronal cultures were fixed in 2.5% glutaraldehyde in 0.1 M phosphate buffer, post-fixed in 2% buffered osmium tetroxide, dehydrated in graded alcohol concentrations and embedded in epoxy resin according to standard protocols. Ultrathin horizontal sections were stained with uranyl acetate and lead citrate and were examined with a transmission electron microscope (EM 906E; Carl Zeiss NTS).

### Protein extraction and western blotting

Cells were lysed in lysis buffer (2% NP40, 10% glycerol (both Sigma), 150 mm NaCl, 50 mm HEPES, 10 mm Na-pyrophosphate, 2 mm Na-orthovanadate, 1 mm NaF (all Carl Roth) and 1 tablet ethylenediaminetetraacetic acid-free complete mini protease inhibitor cocktail (Roche)) on ice prior to ultra-sonication (5 cycles, 75% activity, 5 s). After centrifugation, supernatants were collected and protein contents were estimated using a bicinchoninic acid assay (Thermo Scientific). Unless otherwise stated, 20 µg protein samples were loaded for Sodium dodecyl sulfate–polyacrylamide gel electrophoresis, run in Mini-Protean Tetra Cell gel systems (BioRad) at 120–150 V. Separated proteins were wet-transferred to polyvinylidene fluoride (PVDF) membranes using XCell II Blot modules (Invitrogen). PVDF membranes were blocked in 5% Blotting-Grade Blocker (BioRad) solution (in Tris-buffered saline with Tween-20). Primary antibodies (see Supplementary Material, Table S1) were diluted in blocking solution and incubated overnight at 4°C. Horseradish peroxidase-conjugated secondary antibodies were diluted in blocking solution and incubated for 1–3 h at RT. Blots were developed on Hyperfilms (GE Healthcare) by using Luminol-based chemiluminescent solutions (ECL blotting solution, Amersham) and an automated developer system (Curix60, AGFA l).

### Neurite length, branching points and primary neurite measurements

Neuronal cultures were grown in 24-well plates with glass coverslips for 27 days and then transfected with pEF1-dTomato (gift from F. Perez-Branguli (20) using Lipofectamine 2000 (Invitrogen). The ratio of transfection reagent to DNA was 1:2. Cells were fixed 24–48 h after transfection, stained and then detailed overview images were scanned using the Mosaix software (Zeiss) and a 40× objective on the Observer.Z1 fluorescence microscope (Zeiss). Tracings of individual cells were performed semiautomatically using the NeuronJ plugin of ImageJ. A minimum of 21 cells per line with a total of 96 patient cells and 116 control cells from 4 to 6 wells were analyzed to estimate average neurite lengths, number of branching points and number of primary neurites per cell.

### Axonal transport measurements

Axonal transport was analyzed in neurons of two control (Ctrl-121 and -211) and two SPG4 lines (SPG4-121 and -211) cultured in microfluidic chambers (SND450, Xona Microfluidics, Temecula, CA, USA) for 2 weeks to allow directed and parallel growth of axons and identification of transport directions. Microfluidic chambers were prepared according to the manufacturer's instructions. The microfluidic chambers were assembled and coated with PORN (50 µg/ml)/laminin (5 µg/ml) in DMEM/F12 overnight at 37°C. A total of 80 000 NPCs and 20 000 human astrocytes (ScienCell Research Laboratories, Carlsbad, CA, USA) were seeded on the soma side. In addition, 20 000 human astrocytes were plated to the axonal side of each microfluidic chamber. Cells were cultured in neuronal differentiation medium containing 2% FBS. Mitochondria were visualized by overexpression of Mito-DsRed after lentiviral infection of the soma side at a MOI of 1, 2 days after seeding NPCs. The medium was replaced 48 h after infection. Live-cell imaging was performed on axons in the microgroove area of the microfluidic chambers at stable temperature and balanced CO_2_ conditions using a Nikon Eclipse Ti inverted microscope (Nikon, Duesseldorf, Germany) equipped with a EMCCD camera (model DU-885, Andor Scientific Cameras) and a NIS-Elements AR 3.2 software (Nikon). For live-cell imaging, the culture media was replaced by a live-cell imaging buffer (10 mm HEPES, 144 mm NaCl, 2.5 mm KCl, 10 mm Glucose, 2.5 mmCaCl_2_, 2.5 mm MgCl_2_) with an osmolarity equal to the that of the culture media (309 mmol/kg). Time-lapse recording conditions were setup as one frame per second with a total duration time of 10 min. At least 20 neurites per line were recorded and analyzed. Time-lapse recordings were analyzed by creating kymographs for a single neurite with the time and displacement length of mitochondria using the ImageJ 1.47f software and Multiple Kymograph plugin. Mitochondria transport parameters (speed and transport direction) were obtained from single tracks of moving events on the kymographs, and the number of moving versus stationary events per neurite were calculated. The thresholds for transport events in either anterograde or retrograde direction were considered at a minimum displacement length of ≥5 µm and if the mitochondria were tractable for a minimum of 3.5 min (1/3 of total imaging length).

### Electrophysiology

Whole-cell patch clamp recordings were performed at RT using an EPC 10 amplifier (HEKA electronics) in artificial cerebrospinal fluid bubbled with 95% O_2_ 5% CO_2_ (in mm: 125 NaCl, 3 KCl, 1 CaCl_2_, 1 MgCl_2_, 1.25 NaH_2_PO_4_, 25 NaHCO_3_ and 10 d-glucose; pH 7.4). The internal solution contained (in mm) 4 NaCl, 135 K-gluconate, 3 MgCl_2_, 5 EGTA, 5 HEPES, 2 Na_2_-ATP and 0.3 Na_3_-GTP (pH 7.25).

### Spastin and GFP overexpression

Human SPAST (M1 or M87 isoform), including a C-terminal myc/flag tag, was cloned from the pCMV6-Entry vector (Origene) into the lentiviral pCAG-IRES-GFP vector (gift from I. Verma, Salk Institute, La Jolla, CA, USA) using In-Fusion HD Cloning (Invitrogen). Neuronal cultures were seeded on PORN/laminin-coated glass coverslips in 24-well plates at a density of 40 000–50 000 cells/cm^2^ in neuronal differentiation medium. After 48 h, cells were infected with SPAST or empty GFP control lenitvirus with an MOI of 0.25–0.5. Neuronal cultures were allowed to differentiate for 27 days after seeding, then transfected with pEF1-dTomato and fixed 24 h thereafter. Infected cells were highlighted with anti-GFP staining. Ten to 16 cells per line from 4 to 6 wells were analyzed for the control lines Ctrl-111 and Ctrl-212 and all four SPG4 lines.

### PCR and qRT–PCR

RNA was extracted using the RNeasy Minikit (Qiagen), including on-column DNA digestion. Isolated RNA was quality controlled by visualization in ethidiumbromide containing TAE-agarose gels under UV light. One microgram of RNA was reverse transcribed using the QuantiTect Reverse Transcription kit (Qiagen). PCRs were performed using Taq DNA polymerase in ThermoPol Buffer and dNTP mix (all NEB) with or without 3 mm MgCl_2_ in a C1000 cycler (BioRad). Primers used were (in 3′–5′ direction): nanog-for aaggtcccggtcaagaaacag, Nanog-rev cttctgcgtcacaccattgc, Oct4-for gtgttcagccaaaagaccatct, Oct4-rev ggcctgcatgagggtttct, GAPDH-for tgttgccatcaatgacccctt, GAPDH-rev ctccacgacgtactcagcg, HPRT-for cctggcgtcgtgattagtg, HPRT-rev tcccatctccttcatcacatc, β2M-for gaggctatccagcgtactcc, β2M-rev aatgtcggatggatgaaacc, Katna1-for1 agcactcccttgaaagcgg, Katna1-rev1 gcgttttctaggtcctggtga.

### Statistics

All statistical analyses were performed in GraphPad Prism, version 5.00. For comparisons of the mean between two groups, one-tailed or two-tailed *t*-tests were applied. The groups consisted of the means of each line. One-way analysis of variance followed by Bonferroni's multiple comparison tests were performed when three or more groups were compared. Of note, *χ*^2^ analysis was used to compare parameters of axonal transport. *P*-values ≤0.05 were considered significant (*P≤ 0.05, **P≤ 0.01 and ***≤ 0.001). All data are shown as mean ± standard error mean.

### Supplementary methods

The supplementary methods describe virus production, soma size measurement, spastin fluorescence density measurements, cell death assays and karyotyping.

## SUPPLEMENTARY MATERIAL

Supplementary Material is available at *HMG* online.

## FUNDING

This work was supported by the Tom-Wahlig Foundation Advanced Fellowship. Additional financial support came from the German Federal Ministry of Education and Research (BMBF, 01GQ113), the Bavarian Ministry of Education and Culture, Sciences and Arts in the framework of the Bavarian Molecular Biosystems Reseach Network, For IPS, the Interdisciplinary Center for Clinical Research (University Hospital of Erlangen), the Bavarian Research Foundation (PIZ-180-10), the German Research Association (DFG LA2740/2-1 to A.L.) and from the Helmsley Foundation (to F.H.G. and M.C.N.M.). Funding to pay the Open Access publication charges for this article was provided by the German Federal Ministry of Education and Research (BMBF, 01GQ113).

## Supplementary Material

Supplementary Data
