# Supplementary material for: Gene dosage-dependent rescue of HSP neurite defects in SPG4 patients’ neurons
Source: Hum Mol Genet. 2013 Dec 30;23(10):2527–41. doi: 10.1093/hmg/ddt644 (PMC3990156; doi:10.1093/hmg/ddt644)
Supplement: Supplementary Data [file supp_23_10_2527__index.html]

Gene dosage dependent rescue of HSP neurite defects in SPG4 patients' neurons — Gene dosage-dependent rescue of HSP neurite defects in SPG4 patients’ neurons — Gene dosage-dependent rescue of HSP neurite defects in SPG4 patients’ neurons — Supplementary Data 

# Gene dosage-dependent rescue of HSP neurite defects in SPG4 patients’ neurons

## Supplementary Data

Supplementary Data

**Files in this Data Supplement:**

- Supplementary Data - Docx file
