# Supplementary material for: Gene dosage-dependent rescue of HSP neurite defects in SPG4 patients’ neurons
Source: Hum Mol Genet. 2013 Dec 30;23(10):2527–41. doi: 10.1093/hmg/ddt644 (PMC3990156; doi:10.1093/hmg/ddt644)
Supplement: Supplementary Data [file supp_ddt644_ddt644supp.docx]

MS ID: HMG-2013-D-01319, Havlicek et al

MS Title: **Gene dosage dependent rescue of HSP neurite defects in SPG4 patients’ neurons**

**Supplementary Figures:**


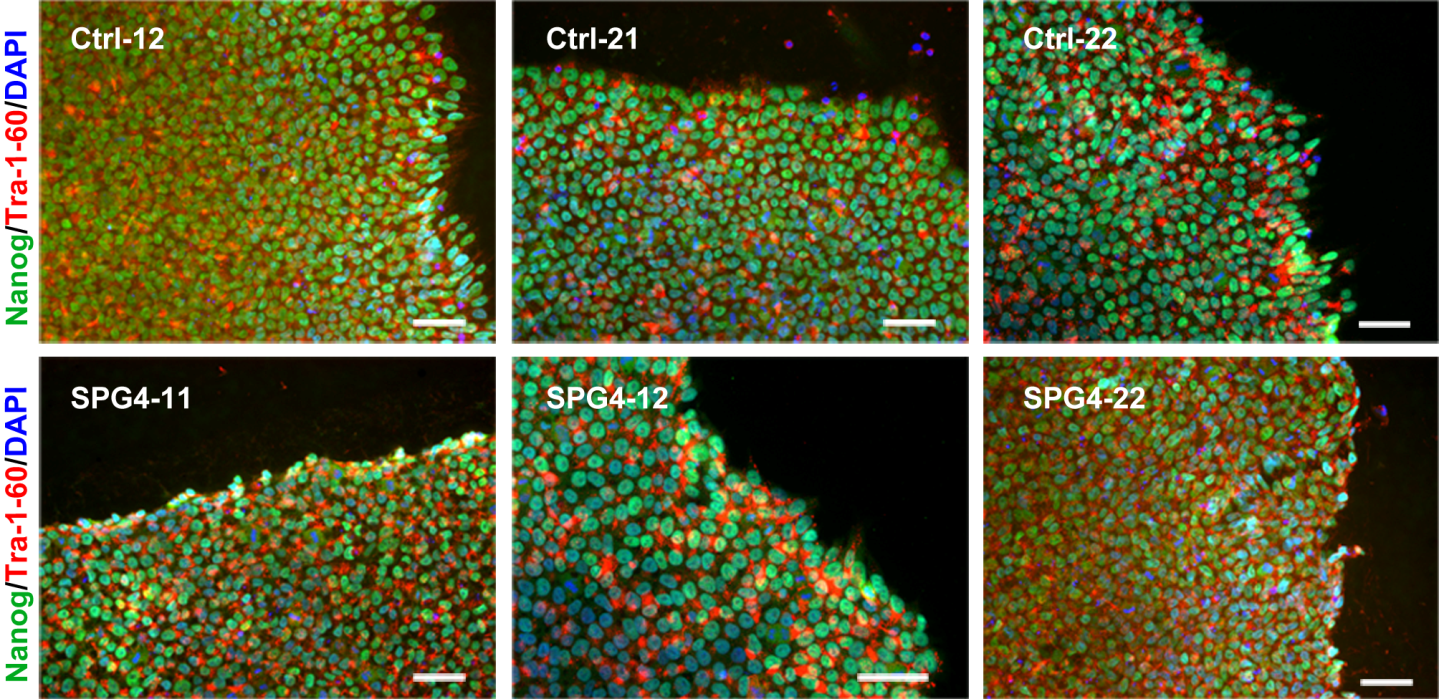


**Supplementary Fig1. Induced pluripotent stem cells (hiPSC) express pluripotency markers.**

Control and SPG4 hiPSCs express endogenous Nanog and Tra-1-60. Scale bars are 50 µm.

**
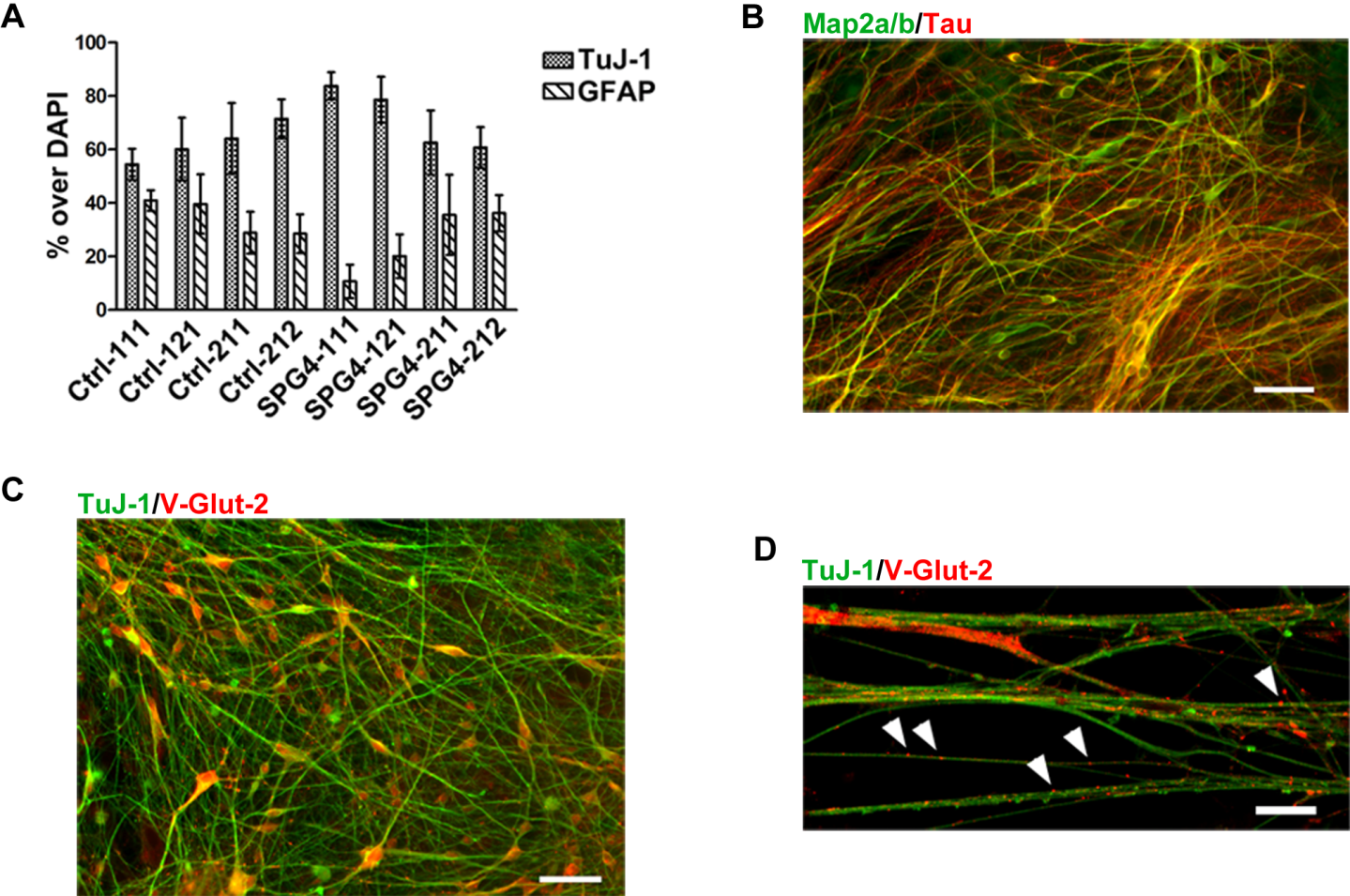
**

**Supplementary Fig2: Characterization of neuronal differentiation**

(A) Control and SPG4 neuronal cultures express neuronal (TuJ-1) and glial markers (GFAP). Amount over DAPI. N = 2 experiments in triplicates. (B) Representative image of Map2a/b and Tau staining of neuronal cultures. (C) Representative overview image of V-Glut-2 and TuJ-1 staining revealing most neuronal cells are glutamatergic. (D) Detailed image of V-Glutv2 staining showing vesicle-like structures along neurites. Scale bars 50 µm in (B) and (C), and 10 µm in (D).

**

**

**Suppl. Fig. 3: Validation of Spastin expression constructs**

M1, M87, M1-R562X, and M87-R562X lentiviral expression constructs were overexpressed in HEK 293T cells (as indicated above the lanes). 15 µg protein lysates were used for Western blot analysis and probed with anti Spastin antibody. Differences in band intensities are due to differences in transfection efficiencies in HEK 293T cells. NT = non transfected control.

**
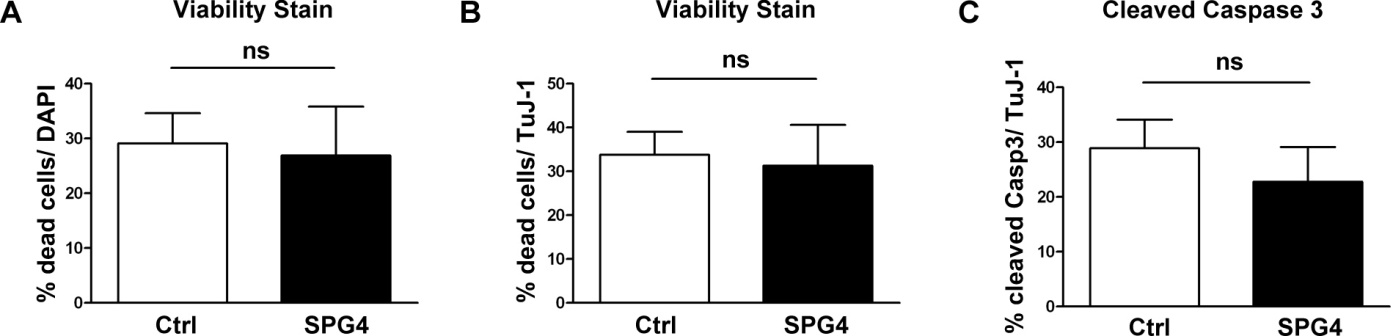
**

**Supplementary Fig4: Estimation of cell death in neuronal cultures**

Total cell death was estimated in neuronal cultures (A) as dead cells/ total DAPI cells, p = 0.831, (B) and dead cells/ TuJ-1+ cells, p = 0.811, using Image-iT® DEAD™ Green viability stain. No difference was observed between control and SPG4 samples. (C) No difference in the amount of apoptotic cells was observed between control and SPG4 neuronal cultures estimated by co-staining of TuJ-1 and cleaved Caspase 3, p = 0.189. N = duplicates of all 8 lines. Data shown as means ± SEM.

**Supplementary Tables:**

| **Target** | **Species** | **Company** | **Application** | **Dilution** |
| --- | --- | --- | --- | --- |
| β3 Tubulin | Ms | Covance | IF | 1:350 |
| β3 Tubulin | Rb | Covance | IF | 1:350 |
| Cleaved Caspase3 | Rb | Cell Signaling | IF | 1:1600 |
| Ctip2 | Rat | Abcam | IF | 1:300 |
| GAPDH | Ms | Calbiochem | WB | 1:15000 |
| Gata4 | Rb | Santa Cruz | IF | 1:200 |
| GFAP | Gt | Abcam | IF | 1:1000 |
| GFP | Chk | Invitrogen | IF | 1:1000 |
| Map2a/b | Ms | Sigma | IF | 1:400 |
| Nanog | Gt | R&D Systems | IF | 1:200 |
| Nestin | Ms | Millipore | IF | 1:300 |
| PSD-95 | Gt | Abcam | IF | 1:400 |
| SMA | Ms | Sigma | IF | 1:400 |
| Sox2 | Rb | Cell Signaling | IF | 1:300 |
| Spastin | Ms | Abcam | WB | 1:2500 |
| Spastin | Rb | Sigma | WB | 1:2000 |
| Spastin | Rb | Sigma | IF | 1:200 |
| Synaptophysin | Ms | Sigma | IF | 1:500 |
| Tau | Gt | Santa Cruz | IF | 1:50 |
| Tubulin, acetylated | Ms | Sigma | IF  WB | 1:1000  1:20000 |
| Tubulin, detyrosinated | Rb | Millipore | IF  WB | 1:1000  1:20000 |
| Tra 1-60 | Ms | Millipore | IF | 1:200 |
| V-Glut2 | Ms | Synaptic System | IF | 1:500 |
| Alexa 488 | Dk | Jackson IR | IF | 1:800 |
| Alexa DyeLight 488 | Dk | Jackson IR | IF | 1:800 |
| Alexa 555 | Dk | Jackson IR | IF | 1:800 |
| Alexa DyeLight 549 | Dk | Jackson IR | IF | 1:800 |
| Alexa 647 | Dk | Jackson IR | IF | 1:800 |
| Alexa DyeLight 649 | Dk | Jackson IR | IF | 1:800 |

Table S1: List of antibodies

Dk: donkey, Ms: mouse, Rb: rabbit, Gt: goat, Chk: chicken, IF: immunofluorescence, WB: Western Blot

**Supplementary Methods:**

*Virus production*

Retro- and lentivirus was packaged in human embryonic kidney (HEK) 293T cells (DSMZ, Germany) grown in Iscove′s Modified Dulbecco’s Media IMDM/Glutamax, and 10% FBS (both Invitrogen). Retroviral plasmids for human Klf4, c-Myc, Oct4, and Sox2 (Takahashi et al., 2007a; Takahashi et al., 2007b) were obtained from Addgene. Per 15-cm plate, the following solution was prepared: 4.6 µg CMV-VSVG, 9.2 µg CMV-gp, 13.8 µg retroviral target construct DNA. For the Lentiviral plasmids (pCAG-SPAST(M1/or M87)-IRES-GFP and pEF1α-IRES-GFP) following solution was prepared: 8.1 µg CMV-MDL, 3.1 µg CMV-RevRSV, 4.1 µg CMV-VSVG, and 12.2 µg target construct. Medium was changed after 6 hours. Viral supernatants were harvested 48 and 62 hours after transfection, filtered through a 0.45-mm SteriCup (Millipore, Billerica, MA, USA) and stored at -80°C.

*Karyotyping*

Standard G-banding chromosome analysis was performed by Cell Line Genetics (Madison) or at the Centre for Human Genetics, Regensburg, Germany.

*Soma size measurements*

Four weeks differentiated neuronal cells were fixed, stained for TuJ-1 and imaged with a 20x objective. Nine images per line were taken randomly from triplicates. Somas were outlined in ImageJ and surface area (= soma size) calculated. A minimum of 100 cells per line from two experiments were analyzed and used to estimate the average soma size.

*Spastin density measurements*

All control and SPG4 neuronal cultures were fixed in 4% PFA after 4 weeks and stained using the SP6C6 Spastin rabbit antibody (Sigma). During the staining all samples were treated simultaneously and with the same antibody master mix. All samples (duplicate wells per line) were imaged using the same exposure time settings (three randomly chosen areas per well). Images were quantified using ImageJ by setting the threshold and pixel size to only measure the average Spastin density in the soma regions on each picture.

*Cell death analyses*

All control and SPG4 neuronal cultures were differentiated for 4 weeks and then either used for viability assays or cleaved Caspase3 assays as follows. For viability assays, the cells were incubated for 30 min at 37°C with Image-iT® DEAD™ Green viability stain (Life Technologies) prior to PFA (4%) fixation and immunofluorescence staining. For cleaved Caspase3 assays, cells were fixed in PFA (4%), washed, incubated in acidic acid: Ethanol (1:2, v:v) for 5 min at -20°C, washed and then used for immunofluorescence staining. In both assays, the cells were stained with TuJ-1 to determine neuronal cells and DAPI to visualize all cells present. Three random images per well (triplicate wells per cell line) were acquired and cell death was estimated by counting manually double positive cells.

*Scholl Analysis of neurite complexity*

Tracings of SPG4 and control neurons were analyzed using the Scholl analysis plugin for ImageJ, version v3.1. 28 control neurons from the lines Ctrl-111 and Ctrl-212 and 28 SPG4 neurons from the lines SPG4-111 and SPG4-212 were analyzed by using the soma as the center. The starting radius was defined as 10 µm followed by 50 µm radii step sizes. The average number of intersections were calculated and plotted linearly against the radius step size.
